# Value of the Leucocyte Count

**Published:** 1907-03

**Authors:** 


### VALUE OF THE LEUCOCYTE COUNT.

Frederic E. Sondern says that his continued daily contact with cases in which the diagnostic aid of the differential leucocyte count is sought strengthens rather than weakens his belief in this valuable factor in surgical diagnosis. It presents diagnostic and prognostic data at a time when the clinical picture may be confusing. While it is true, continues the writer, that pitfalls and errors surround this as they do all other diagnostic methods, and while it is of use to only the discriminating clinician who learns to apply it, still nobody can gainsay the fact that it fills a place occupied by nothing else.—Medical Record, December 22, 1906.

